# Cooperation of endothelin-1 signaling with melanosomes plays a role in developing and/or maintaining human skin hyperpigmentation

**DOI:** 10.1242/bio.011973

**Published:** 2015-09-04

**Authors:** Daiki Murase, Akira Hachiya, Mamiko Kikuchi-Onoe, Rachel Fullenkamp, Atsushi Ohuchi, Takashi Kitahara, Shigeru Moriwaki, Tadashi Hase, Yoshinori Takema

**Affiliations:** 1 Biological Science Laboratories, Kao Corporation, Haga, Tochigi 321-3497, Japan; 2 Biological Science Americas Laboratory, Kao Corporation, Cincinnati, OH 45214, USA; 3 Research and Development Global, Kao Corporation, Sumida-ku, Tokyo 131-8501, Japan

**Keywords:** Endothelin-1, Hyperpigmentation, Senile lentigo, Melanin, Melanosome

## Abstract

Skin hyperpigmentation is characterized by increased melanin synthesis and deposition that can cause significant psychosocial and psychological distress. Although several cytokine-receptor signaling cascades contribute to the formation of ultraviolet B-induced cutaneous hyperpigmentation, their possible involvement in other types of skin hyperpigmentation has never been clearly addressed. Since our continuous studies using skin specimens from more than 30 subjects with ethnic skin diversity emphasized a consistent augmentation in the expression of endothelin-1 (ET-1) and its receptor (Endothelin B receptor, ET-B) in hyperpigmented lesions, including senile lentigos (SLs), the precise function of ET-1 signaling was investigated in the present study. In line with previous studies, ET-1 significantly induced melanogenesis followed by increases in melanosome transport in melanocytes and in its transfer to keratinocytes while inhibition of ET-B function substantially depressed melanogenic ability in tissue-cultured SLs. Additionally, in agreement with a previous report that the formation of autophagosomes rather than melanosomes is stimulated according to starvation or defective melanosome production, ET-1 was found to remarkably augment the expression of components necessary for early melanosome formation, indicating its counteraction against autophagy-targeting melanosome degradation in melanocytes. Despite the lack of substantial impact of ET-1 on keratinocyte melanogenic functions, the expression of ET-1 was enhanced following melanosome uptake by keratinocytes. Taken together, our data suggest that ET-1 plays a substantial role in the development and/or maintenance of skin hyperpigmentation in reciprocal cooperation with increased melanosome incorporation.

## INTRODUCTION

Hyperpigmented age spots, referred to as senile lentigos (SLs) when diagnosed by medical doctors, are characterized by enhanced melanin accumulation and the elongation of epidermal rete ridges (Unver et al*.*, 2006). They are common forms of skin hyperpigmentation due to both long-term sun-exposure and chronological aging. Such hyperpigmentation is also thought to be related to the existence of uneven skin tones often observed on sun-exposed areas. Regardless of the significant psychosocial distress associated with age spots, little is known about the detailed mechanisms responsible for them, except for ultraviolet B (UVB)-induced pigmentation, despite the fact that many researchers have tried to identify the melanogenic stimulatory factor(s) involved.

In the course of UVB-induced pigmentation, three major steps in the epidermis, melanocyte proliferation, activation of melanin synthesis and melanosome transfer to keratinocytes, have been reported to be responsible for the increased melanogenesis (Okazaki et al*.*, 1976; Rosdahl and Szabo, 1978; Imokawa and Mishima, 1982; Mishima and Imokawa, 1983). Especially for the first two steps, several complicated networks comprised of paracrine and autocrine cytokines secreted by keratinocytes and by melanocytes, respectively, are known to play important roles in regulating melanogenesis through their corresponding receptors, whose expression is also regulated by various cytokines (Halaban et al*.*, 1988; Imokawa et al*.*, 1992, 1996, 2000; Yohn et al*.*, 1993; Schauer et al*.*, 1994; Abdel-Malek et al., 1995; Sirsjö et al*.*, 1996; Wintzen and Gilchrest, 1996; Roméro-Graillet et al*.*, 1997). Among such cytokine-receptor signaling cascades, α-melanocyte stimulating hormone (αMSH) and its receptor, melanocortin 1 receptor (MC1R), endothelin-1 (ET-1) and its receptor (Endothelin B receptor, ET-B) and stem cell factor (SCF) and its receptor (KIT), have been shown to substantially contribute to UVB-induced melanosis (Imokawa et al., 1992, 1995, 1997; Lerner and McGuire, 1961; Bolognia et al*.*, 1989; Chakraborty et al*.*, 1996; Hachiya, et al., 2001) apart from the verification of the indispensable role of SCF signaling pathway in hair pigmentation using its neutralizing antibody (Hachiya, et al., 2009). In addition to UVB-induced pigmentation, all three signaling pathways noted above have also been reported to be involved in the formation of SLs (Kadono et al., 2001; Hattori et al., 2004; Motokawa et al., 2005; Murase et al., 2009). The ET-1/ET-B signaling pathway has also been suggested to be involved in the development and/or maintenance of another type of persistent hyperpigmentation, seborrheic keratosis, a benign epidermal tumor with increased pigmentation (Teraki et al., 1996; Manaka et al., 2001). On the other hand, dysfunction of these pathways was found to cause hypopigmentation such as vitiligo (Kitamura, et al., 2004).

Besides the development and/or maintenance of hyperpigmentation, the mechanisms underlying ethnic skin color variation have also been examined, whereas tyrosinase activity has been reported to closely correlate with melanin content, resulting in its critical contribution to skin pigmentation (Iozumi et al., 1993). In the process of melanin biosynthesis, the rate-limiting enzyme activity of tyrosinase has been suggested to cooperate with a series of other tyrosinase-related melanogenic enzymes, including dopachrome tautomerase and tyrosinase-related protein-1 (Tyrp1), mutations of which cause hypopigmentation or diluted skin color (Searle, 1990; Park et al., 1993; del Marmol and Beermann, 1996; Boissy et al., 2006). In addition to the tyrosinase-related enzymes, mutations in genes encoding melanosome transport-related molecules, such as Myosin Va, RAB27A and SLAC-2A, have been also shown to cause a human pigment disease, Griscelli syndrome, which is mostly characterized by diluted skin and hair color (Ménasché et al., 2003; Van Gele et al., 2009). These findings encouraged us to investigate the function of melanosome transport within melanocytes in modulating skin color variations and consequently led us to reveal the essential role of RAB27A, a small Ras-like GTPase belonging to the Rab family, which is in charge of various types of membrane transport (Fukuda, 2008; Stenmark, 2009), in the determination of human ethnic skin color (Yoshida-Amano et al., 2012). We demonstrated the correlation of RAB27A expression to skin color intensity in human skin substitutes. On the other hand, the ability of melanosome transfer from melanocytes to keratinocytes has been also suggested to determine distinct ethnic skin colors. For example, protease-activated receptor-2, a seven transmembrane G-protein-coupled receptor, which is dominantly in charge of phagocytosis in keratinocytes, has been demonstrated to be expressed at a higher level in darker skin compared with lighter skin (Sharlow et al., 2000; Babiarz-Magee et al., 2004). In addition to melanosome transport and transfer, we recently proposed that epidermal melanosome degradation mainly driven by autophagy, an intracellular process whereby the cytosol and organelles are sequestered within double-membrane–bound autophagosomes that subsequently deliver their contents to lysosomes/vacuoles for degradation (Seglen and Bohley, 1992; Yoshimori, 2004; Levine and Klionsky, 2004; Mizushima, 2007), also plays a role in determining ethnic skin color diversity (Murase et al., 2013). Concisely, following the confirmation of the significantly higher autophagic activity in Caucasian skin-derived keratinocytes compared to African American skin-derived keratinocytes, inhibition or activation of autophagy substantially darkened or lightened the skin color, respectively, via regulating melanosome degradation in keratinocytes in the previous study. Related to the involvement of autophagy in skin pigmentation, regulators of autophagy have been recently hypothesized to play different roles in both the biogenesis of melanosomes and their destruction in melanocytes (Ho and Ganesan, 2011).

As mentioned above, various mechanisms have been considered to be involved in the increased melanogenesis that occurs in different types of skin hyperpigmentation. Given that extremely consistent explanations have been proposed for the cytokine-receptor signaling cascades, especially for the ET-1/ET-B signaling pathway, in the formation and/or maintenance of hyperpigmentation, it would be reasonable to hypothesize that the destiny of hyperpigmentation substantially relies at least on that pathway. Therefore, in the present study, the impact of the signaling pathways discussed above on the induction and/or maintenance of skin hyperpigmentation and their mechanisms were comprehensively reexamined using various approaches including existing hyperpigmented skin specimen's utilization.

## RESULTS

### Significantly enhanced expression of ET-1 and its receptor is consistently observed in hyperpigmented skin areas compared with even-toned skin areas

**Fig. 1.**
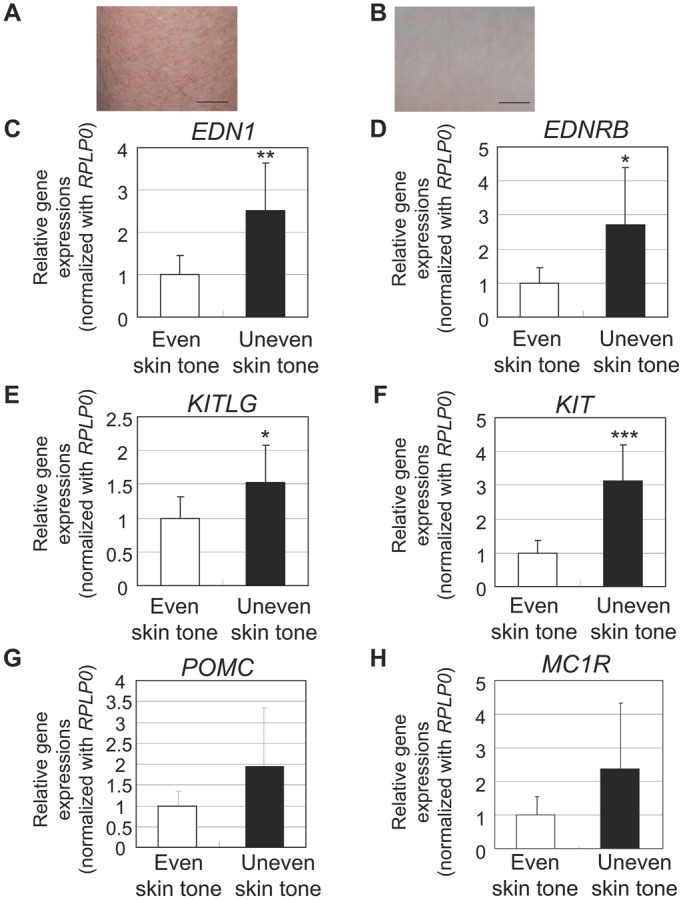
**Increased expression of melanogenic paracrine cytokines and their receptors in uneven-toned skin.** (A) Clinical appearance of uneven–toned skin on the upper arm of a Caucasian female subject. Scale bar=1 cm. (B) Clinical appearance of even–toned skin on the upper arm of another Caucasian female subject. Scale bar=1 cm. mRNA transcript levels of *EDN1* (C), *EDNRB* (D), *KITLG* (E), *KIT* (F), *POMC* (G) and *MC1R* (H) were measured using *Taq*Man real-time PCR in epidermal sheets obtained from even-toned and uneven-toned skin. mRNA transcript levels were normalized to *RPLP0* (ribosomal protein large P0). Relative amounts of each mRNA transcript in unevenly pigmented skin are expressed as a ratio against even-toned skin. Values represent means±s.d. from eight subjects. ***P<0.001; **P<0.01; *P<0.05 (Student's *t*-test).

**Fig. 2.**
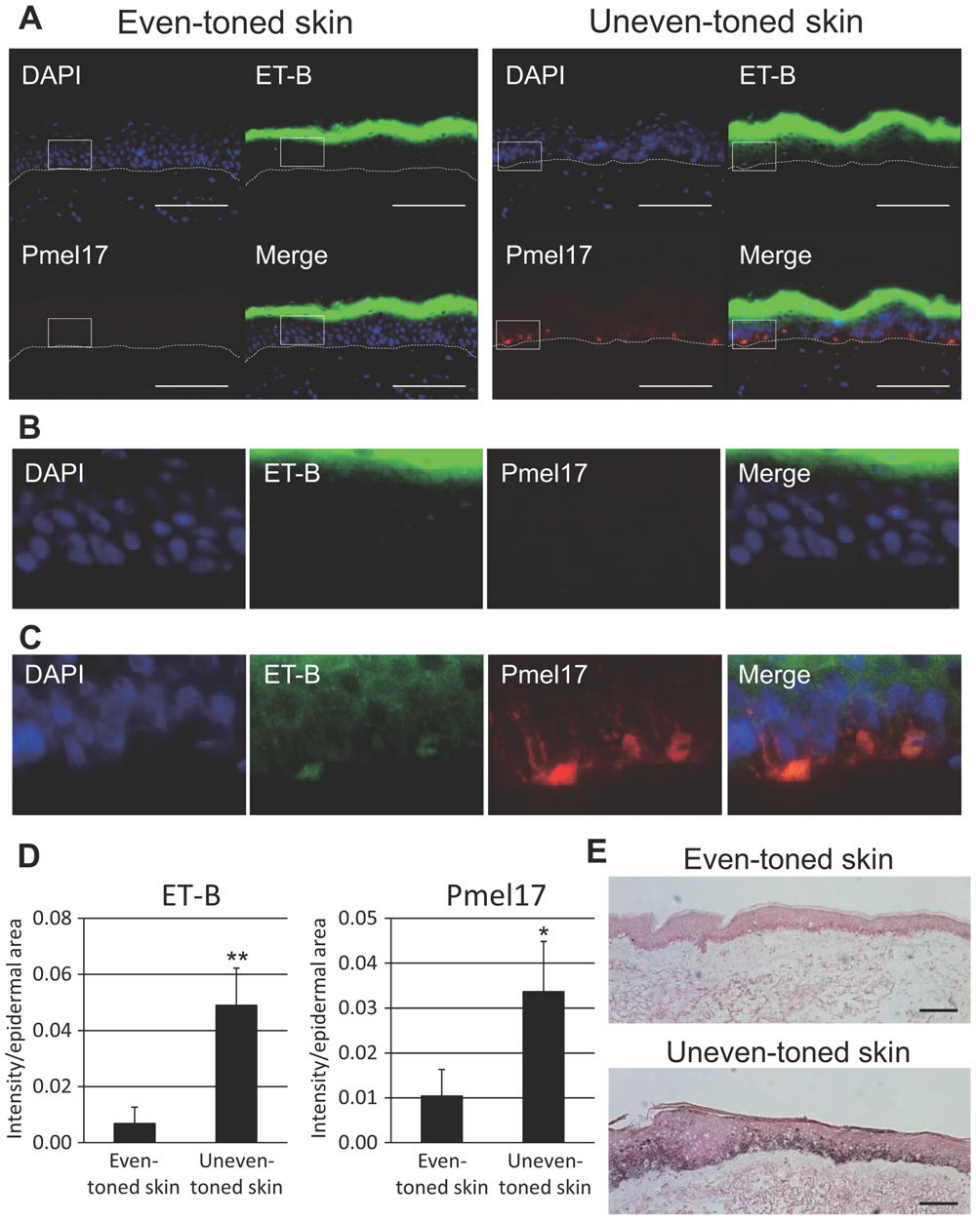
**Increased expression of melanogenic proteins in unevenly pigmented skin.** (A) Immunohistological analysis of ET-B (green) and Pmel17 (red) proteins in uneven-toned skin compared to even-toned skin. Merged images with nuclear staining (4′6-diamidino-2-phenylindole, DAPI; labeled in blue) are also shown. Scale bar=100 µm. (B) Magnified images from the white boxes in the original images of even-toned skin. (C) Magnified images from the white boxes in the original images of uneven-toned skin. (D) The intensity of each signal was normalized with epidermal area using ImageJ analysis software. Left panel, ET-B; right panel, Pmel17. Values represent means±s.d. from at least four different subjects. **P<0.01; *P<0.05 (Student's *t*-test). (E) Fontana–Masson staining of even- and uneven-toned skin is displayed. Scale bars=100 µm.

Although genetic and cellular studies have suggested that *MC1R* polymorphisms contribute to the differences in UV sensitivity and in hair and skin color intensity in several ethnic groups (Scott et al., 2001; Sturm, 2009), little is known about which cytokine-receptor signaling cascade(s) is most involved in the induction and/or maintenance of skin hyperpigmentation. Therefore, in order to reexamine the impact of the ET-1/ET-B, SCF/KIT and αMSH/MC1R signaling pathways on hyperpigmentation, more than 30 subjects with ethnic skin diversity (Caucasian, Hispanic and Asian) were recruited and skin biopsies with or without hyperpigmentation were taken only from sun-exposed areas for comparison. Real-time RT-PCR analysis indicated a significantly higher mRNA expression of ET-1 (*EDN1*) and ET-B (*EDNRB*) in SLs, which is consistent with previous reports (Kadono et al., 2001; Murase et al., 2009) (supplementary material Fig. S1A,B and Fig. S2A,B). On the other hand, mRNA expression levels of SCF (*KITLG*) and the precursor of αMSH, pro-opiomelanocortin (*POMC*), did not differ significantly although mRNA levels of their receptors (*KIT* and *MC1R*, respectively) in SLs were significantly higher than in peripheral areas (supplementary material Fig. S1C-F). In addition, a significantly enhanced expression of *EDN1* and *EDNRB* was consistently observed even in unevenly pigmented skin (lighter discoloration shown in Fig. 1A) compared with evenly pigmented skin (Fig. 1B-D). Further, immunohistochemical analyses confirmed significantly enhanced expressions of ET-B and Pmel17 along with increased melanin deposition in uneven-toned skin than in even-toned skin, which is consistent with the function of ET-1/ET-B signaling in the induction and/or maintenance of cutaneous hyperpigmentation (Fig. 2A-E). In contrast to ET-1 signaling, the impact of SCF/KIT signaling on the augmented melanogenesis was detected only in unevenly pigmented skin due to the significantly higher transcript expression levels whereas no significant increase in *KITLG* mRNA expression was observed in SLs (supplementary material Fig. S1C,D; Fig. 1E,F). No significantly enhanced *POMC* expression was found in SLs or in uneven-toned skin despite the significantly higher expression of its receptor, *MC1R*, in SLs (supplementary material Fig. S1E,F; Fig. 1G,H).

### Effect of ET-1/ET-B signaling pathway on NHEMs but not on NHEKs for the acceleration of melanogenesis

**Fig. 3.**
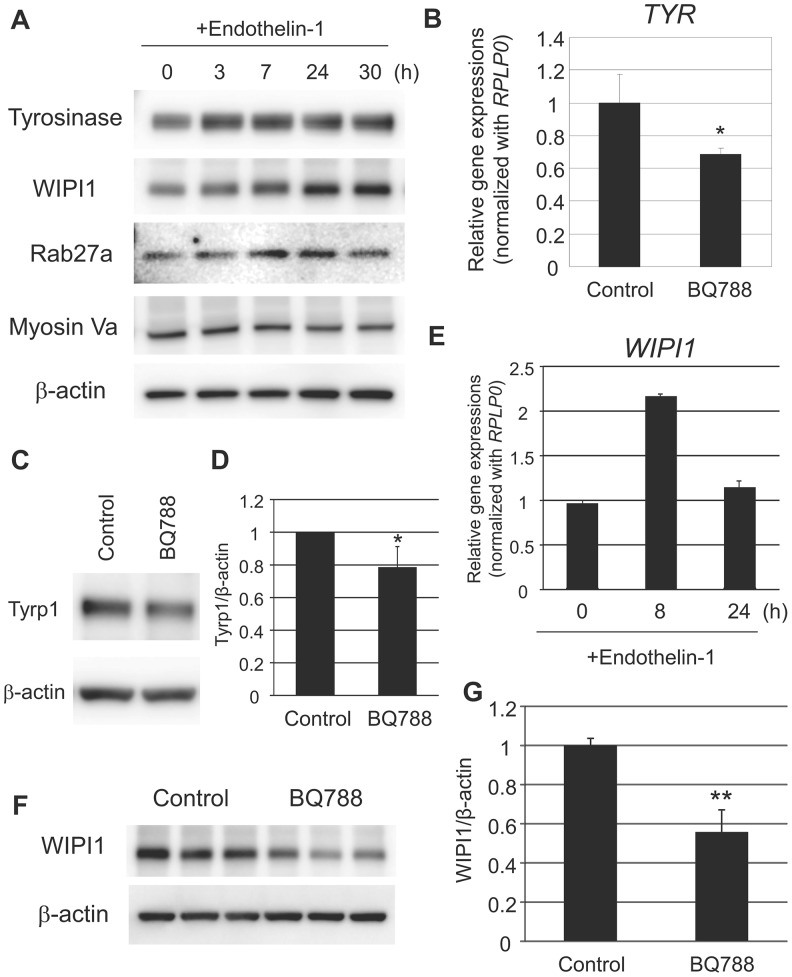
**Substantial role of ET-1 and its receptor signaling in melanogenesis in melanocytes *in vitro* and in uneven-toned skin *ex vivo*.** (A) NHEMs were incubated with 10 nM ET-1 for the indicated times. Cells were then harvested for western blotting analysis using WIPI1-, Tyrosinase-, Rab27A- or Myosin Va-specific antibodies. β-actin was used as a loading control. (B) Uneven-toned skin derived from either the forearm or the face of Caucasian female subjects were divided into two pieces and were subjected to tissue culture with or without 1 µM BQ788. After 72 h of treatment, whole skins were used for gene expression analysis with *Taq*Man real-time PCR. Tyrosinase (*TYR*) mRNA transcript levels were normalized to *RPLP0*, and its relative amount in BQ788-treated skins is indicated as a ratio against the BQ788-untreated control. *P<0.05 (Student's *t*-test). (C) Unevenly pigmented skins were prepared as noted above and were then subjected to tissue culture with or without 1 µM BQ788. After 72 h of treatment, whole skins were used for western blotting analysis using a Tyrp1-specific antibody. β-actin was used as a loading control. (D) Relative intensity of each band was assessed after normalization against β-actin, and its relative amount in BQ788-treated skins is indicated as a ratio against the BQ788-untreated control. Values represent means±s.d. from five subjects. *P<0.05 (Student's paired *t*-test). (E) NHEMs were treated with 10 nM ET-1 for the indicated times. Cells were collected for gene expression analysis with *Taq*Man real-time PCR. *WIPI1* mRNA transcript levels were normalized to *RPLP0.* (F) NHEMs were treated with 1 µM BQ788 in the presence of 10 nM ET-1 for 24 h. Cells were harvested for western blotting analysis using a WIPI1-specific antibody. The blot was re-probed with a β-actin antibody to check the loading amount. (G) Relative intensity of each band was assessed after normalization against β-actin. Values represent means±s.d. from three samples. **P<0.01 (Student's *t*-test).

The reconfirmation of the consistently higher expression levels of *EDN1* and *EDNRB* mRNAs in hyperpigmented skin compared to sun-exposed control areas without hyperpigmentation led us to reinvestigate in more detail how the ET-1/ET-B signaling pathway stimulates melanogenesis. First, a reinvestigation of the function of the aforementioned signaling pathway in melanocytes was initiated and the treatment of NHEMs with ET-1 was confirmed to enhance the expression of tyrosinase in agreement with previous studies (Imokawa et al*.*, 1992, 1996, 2000; Yohn et al*.*, 1993) (Fig. 3A). Consistently, the expression of tyrosinase and Tyrp1 was significantly suppressed at the mRNA or protein levels in cultured hyperpigmented skin in the presence of an ET-B antagonist, BQ788, even for 3 days (Fig. 3B-D), verifying the essential role of the ET-1/ET-B signaling pathway in the maintenance of hyperpigmentation.

Following that analysis of the impact of ET-1/ET-B signaling on the expression of conventional melanogenic regulators, its effect on melanosomal regulators was also examined. It has been reported that WIPI1-depleted cells accumulate stage I melanosomes but lack stage III-IV melanosomes (Ho et al., 2011). On the other hand, knockdown of *RAB27A* has been suggested to significantly diminish epidermal melanin content in human skin substitutes *in vivo* after treatment with a lentivirus-derived shRNA in concert with its expressional difference observed between African American and Caucasian skins (Yoshida-Amano et al., 2012). RT-PCR and western blotting analyses demonstrated that treatment with ET-1 enhanced the expression of WIPI1 and RAB27A at the mRNA and/or protein expression levels in NHEMs and that inhibition of ET-B significantly reduced WIPI1 protein expression in the presence of ET-1 (Fig. 3A,E-G). In contrast to the increase in RAB27A expression, no significant change in Myosin Va expression was observed in ET-1-treated cells (Fig. 3A).

**Fig. 4.**
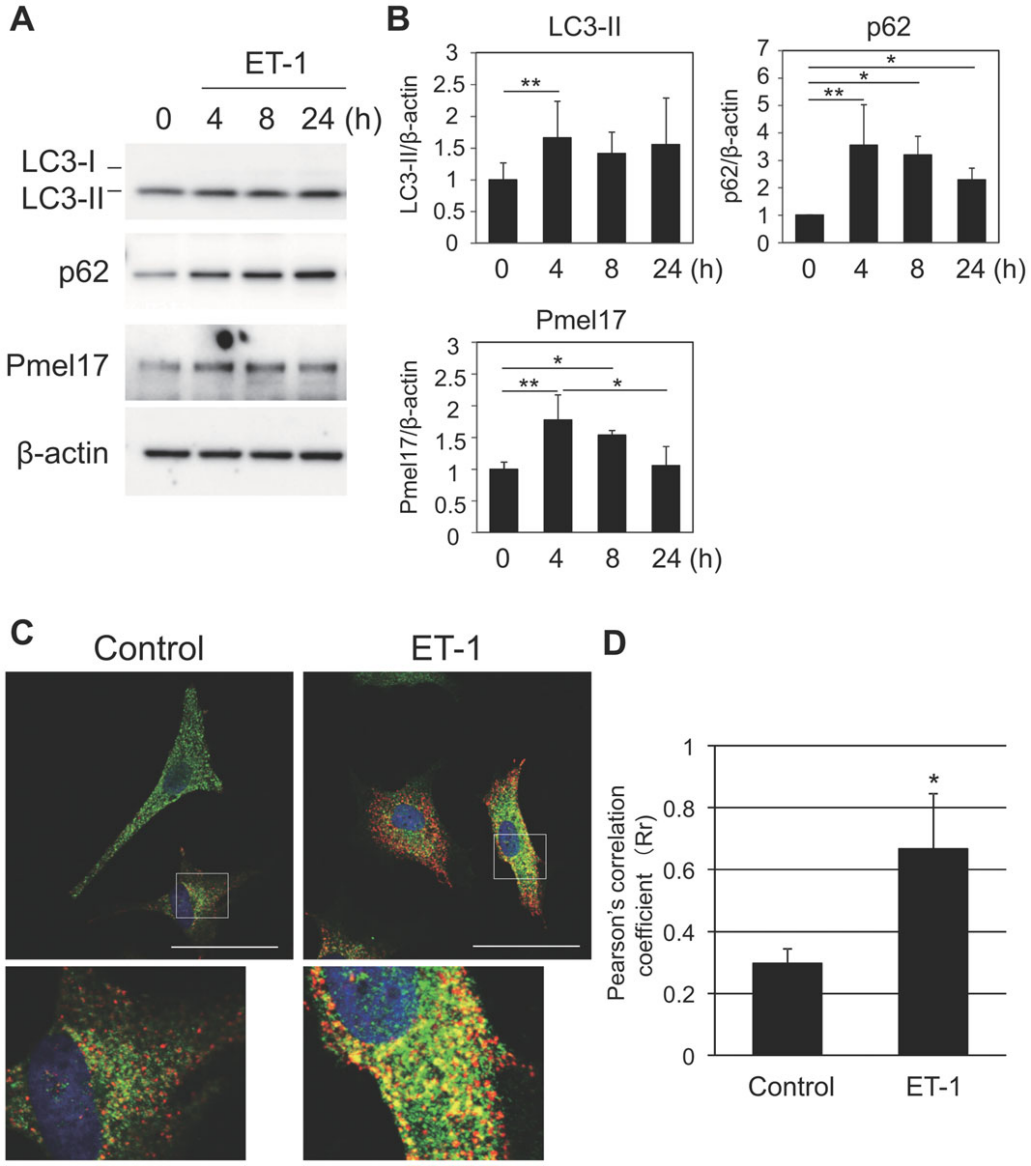
**Acceleration of melanosome generation by ET-1 counteracting autophagy-driven degradation in melanocytes.** (A) NHEMs were treated with 10 nM ET-1 for the indicated times. Cells were harvested for western blotting analysis using LC3-, p62- or Pmel17-specific antibodies. The blots were then re-probed with a β-actin-specific antibody for a loading control. (B) Relative intensity of each band normalized to β-actin was expressed as a ratio against the control. Values represent means±s.d. from three different experiments. **P<0.01; *P<0.05 (ANOVA, Holm test). (C) NHEMs were cultured with or without 10 nM ET-1 for 24 h and were then subjected to immunofluorescence staining with an anti-p62 antibody (green) and an anti-Pmel17 antibody (red). Merged images with nuclear staining (DAPI) are shown. The colocalization of p62 and Pmel17 is observed as yellow dots. Scale bar=50 µm. (D) The values of colocalization of p62 and Pmel17 were quantified and expressed as Pearson's correlation coefficient by ImageJ analysis software. Values represent means±s.d. from four samples. *P<0.05 (Student's *t*-test).

Furthermore, the impact of ET-1 on the autophagy regulators was subsequently evaluated according to a paper that hypothesized that autophagy regulators play different roles in both the biogenesis of melanosomes and in melanosome destruction (Ho and Ganesan, 2011). In line with that report, the expression of several autophagy regulators, such as LC3-II and p62, was significantly increased following treatment with ET-1 accompanied by a significant enhancement of Pmel17 expression (Fig. 4A,B). In addition, ET-1 significantly augmented the co-localization of p62 with the early melanosome marker Pmel17 (Fig. 4C,D). These findings indicate that ET-1 accelerates the synthesis of early melanosomes in melanocytes, which counteracts the autophagy-targeting melanosome degradation in melanocytes.

**Fig. 5.**
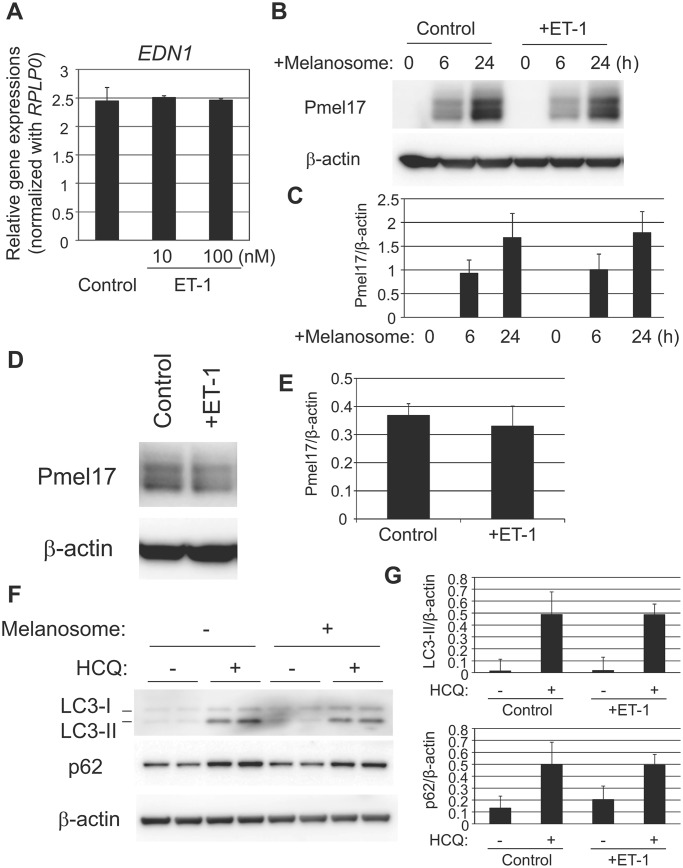
**No substantial impact of ET-1 on melanogenic function in keratinocytes in an autocrine manner.** (A) NHEKs were treated with 10 nM ET-1. After incubation for 24 h, total RNA was isolated from the cells and levels of mRNA transcripts were analyzed by quantitative RT-PCR using *Taq*Man Gene Expression Assays. The mRNA level of *EDN1* was normalized to *RPLP0*. (B) NHEKs were treated with isolated melanosomes with or without 10 nM ET-1 for the indicated times. Cells were then harvested for western blotting analysis using a Pmel17-specific antibody. The blot was re-probed with a β-actin-specific antibody as a loading control. (C) Relative intensity of each band was normalized with that of β-actin. Values represent mean±s.d. from three independent experiments. (D) NHEKs were incubated with isolated melanosomes for 24 h. After removal of non-incorporated melanosomes by washing with PBS, cells were cultured for an additional 24 h with or without 10 nM ET-1. Cells were then subjected to western blotting analysis using a Pmel17-specific antibody. β-actin was used as a loading control. (E) Relative intensity of each band was normalized with that of β-actin. Values represent mean±s.d. from three different experiments. (F) NHEKs were incubated with isolated melanosomes for 24 h. After removal of non-incorporated melanosomes by washing with PBS, cells were cultured with or without 10 µM hydroxychloroquine (HCQ). Twenty-four hours after the treatment, cells were harvested for western blotting analysis using LC3- or p62-specific antibodies. β-actin was used as a loading control. (G) Relative intensity of each band was normalized with that of β-actin. Values represent mean±s.d. from three different experiments. Top panel, LC3-II; bottom panel, p62.

**Fig. 6.**
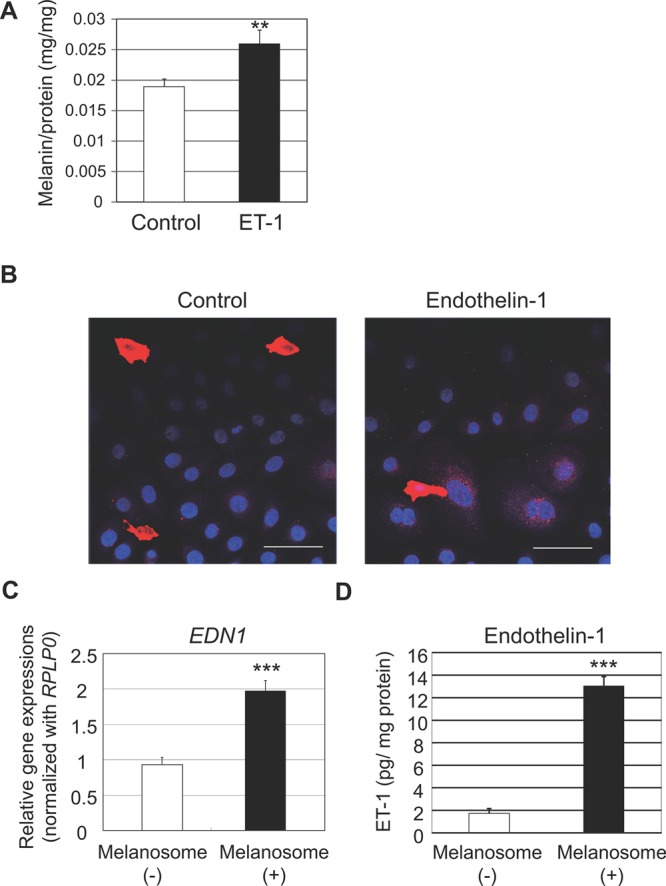
**Reciprocally harmonized interaction between ET-1-induced melanogenesis in melanocytes and melanosome-stimulated ET-1 synthesis in keratinocytes.** (A) NHEMs were co-cultured with NHEKs in the presence or absence of 10 nM ET-1 for 7 days. Cells were then collected for the quantification of cellular melanin content. Values represent means±s.d. from three samples. **P<0.01 (Student's *t*-test). (B) NHEMs were co-cultured with NHEKs in the presence or absence of 10 nM ET-1 for 3 days. Cells were then fixed and subjected to immunofluorescence staining with a Pmel17-specific antibody. Merged images with nuclear staining (DAPI) are shown. Scale bar=50 µm. (C) NHEKs were treated with melanosomes isolated from MNT-1 cells. After incubation for 24 h, total RNA was isolated from the cells and levels of mRNA transcripts were analyzed by quantitative RT-PCR using *Taq*Man Gene Expression Assay. *EDN1* mRNA expression levels were normalized to *RPLP0*. Values represent means±s.d. from three samples. ***P<0.001 (Student's *t*-test). (D) NHEKs were treated with melanosomes isolated from MNT-1 cells. After incubation for 72 h, conditioned media were collected and quantified for levels of ET-1 using ELISA. Values represent means±s.d. from three samples. ***P<0.001 (Student's *t*-test).

In parallel to investigating the effect of ET-1/ET-B signaling on melanocytes, its impact on keratinocytes was also examined since keratinocytes have been reported to express endothelin receptors (Ahn et al., 1998). Alterations in ET-1 synthesis, melanosome incorporation and autophagic melanosome degradation were assessed in the presence of ET-1 but no significant changes were observed, which is consistent with a recent paper reporting that ET-1 knockout has a negligible impact on epidermal proliferation and differentiation (Hyter et al., 2013) (Fig. 5A-E). In line with the increased expression of melanosomal regulators in melanocytes, a comparable significant increase in melanin content was also observed in co-cultures of NHEKs and NHEMs (Fig. 6A), indicating a role for regulators engaged in melanosome transport in melanocytes, and possibly in subsequent melanosome transfer from melanocytes to keratinocytes, in the increase of melanin content induced by treatment with ET-1.

### Reciprocal function of melanosomes to accelerate the synthesis of ET-1 in NHEKs resulting in their accumulation

Consistent with the significant enhancement of melanin content, the co-culture of NHEKs with NHEMs in the presence of ET-1 enhanced the deposition of melanosomes around nuclei in NHEKs compared to co-culture in the absence of ET-1 (Fig. 6B). This finding encouraged us to investigate the effect of increased incorporation of melanosomes on keratinocyte melanogenic activities. Although no changes were observed in melanosome phagocytic activity (data not shown) or autophagic activity (Fig. 5F,G) in NHEKs after the application of isolated melanosomes, their significant increase in ET-1 synthesis in contrast to no changes in the expression of SCF and αMSH (data not shown) was interesting (Fig. 6C,D). These data suggested a reciprocally harmonized interaction between ET-1-induced melanogenesis in melanocytes and melanosome-stimulated ET-1 synthesis in keratinocytes in accordance with the ET-1/ET-B signaling pathway that maintains hyperpigmentation.

## DISCUSSION

Skin aging has been shown to result from a combination of the effects of chronological factors (intrinsic aging) and of environmental ones (extrinsic aging). Intrinsic and extrinsic aging are thought to result from an accumulation of metabolic oxidative stress to the skin microstructure and from cutaneous exposure to UV radiation and environmental pollutants, respectively (Fisher et al., 2002; Chung et al., 2003; Baumann, 2007; Sherratt et al., 2010). In relation to extrinsic aging, chronic exposure to UV radiation has been suggested to mainly cause skin damage known as photodamage, which is characterized by wrinkling, sagging and hyperpigmentation that is usually diagnosed as SLs (Hölzle, 1992). Hyperpigmentation has continued to present clinical management challenges for dermatologists accompanied by the most common complaints in patients regarding skin color problems despite the availability of multiple treatments for improvement (Desai, 2014). This situation encouraged us to examine the causative mechanism(s) from the viewpoint of cytokine signaling engaged in UVB-induced pigmentation according to the many similarities between UVB-induced melanosis and SLs in terms of their clinical and histochemical features (Hattori et al., 2004). Indeed, the recent report suggests that UV-induced long-lasting pigmentation seems to have similar features to other forms of hyperpigmentation, e.g. SLs due to the fact that repeated UV exposure results in an impaired ability to remove melanin in epidermal keratinocytes (Coelho et al., 2015). In the current study, we emphasized, using more than 30 human skin samples, that the stimulated ET-1 signaling substantially contributes to the development and/or maintenance of skin hyperpigmentation in a reciprocal manner with melanosomes derived from melanocytes that accelerate ET-1 synthesis in keratinocytes.

One of the most important issues addressed in this study refers to how the up-regulated ET-1 signaling impacts the hyperpigmentation progress and/or maintenance. It has been reported that SLs are characterized by numerous brown and tan freckles of varying size that appear on the backs of the hands, forearms and faces of elderly people (Jackson, 2001). We considered that SLs are outstanding pigmented spots even against a sun-irradiated background and we were motivated to reexamine their causal signaling pathways. We characterized that by collecting skin samples only from sun-exposed regions and by comparing the expression levels between hyperpigmented spots and their proximal sun-irradiated regions without intense pigmentation. Given that the expression of ET-1 and ET-B, but not the other two interesting pairs of ligands and receptors (αMSH/MC1R and SCF/KIT), was confirmed to be concomitantly increased in SLs compared to their adjacent sun-exposed control areas, it was reasonable to hypothesize that ET-1 signaling plays a substantial role in developing and/or maintaining hyperpigmentation. Consistent with a published paper (Imokawa et al., 1996), the effect of ET-1 signaling to stimulate the expression of melanogenic factors, including tyrosinase and Tyrp1, was verified by administering an ET_B_R inhibitor to tissue-cultured hyperpigmented skins. Subsequently, experiments using treatment of NHEMs with ET-1 and/or an ET-B inhibitor also confirmed that ET-1 signaling impacts the expression of WIPI1 and RAB27A, indicating the contribution of ET-1 signaling to melanosome maturation and to their subsequent transport in melanocytes. Furthermore, consistent with a study proposing that the formation of autophagosomes rather than melanosomes is prioritized under conditions of starvation or defective melanosome production (Ho and Ganesan, 2011), ET-1 stimulation was shown to drive the initiation of melanosome formation in cultured melanocytes, suggesting the counteraction against autophagy-targeting melanosome degradation in melanocytes. Since the melanogenic effects of ET-1 on keratinocytes with endothelin receptors in an autocrine manner (Ahn et al., 1998), such as ET-1 production, melanosome incorporation and/or inhibition of autophagic melanosome degradation, were not found, which was consistent with previous report (Hyter et al., 2013), our data together suggest that the activated ET-1 signaling characterized by the significantly increased expression of both ET-1 and ET-B mainly contributes to the melanogenic machineries in melanocytes, resulting in the formation of localized brownish and tanned patches of skin compared with their peripheral regions.

It was also of interest to explore the contribution of the consequent products (melanins or melanosomes) to the ability of recipients (keratinocytes) that secrete ET-1 to melanocytes from the viewpoint of a harmonized manner in order to additively and/or synergistically enhance melanogenesis. It has been suggested that the UVB-induced increase in eumelanin production above constitutive levels occurs in epidermal melanocytes but not in extracutaneous (e.g. uveal tract) melanocytes (Li et al., 2006). Further, the increased epidermal pigmentation has been considered to provide several potential adaptive advantages to hairless hominids, beyond protection against UV exposure, including camouflage, sexual display, free radical absorber, neuroendocrine functions and cutaneous innate immunity (Jablonski and Chaplin, 2000; Parra, 2007; Schallreuter et al., 2008; Takeda et al., 2007; Mackintosh, 2001). These proposed functions of increased melanin production generally indicate that such a specialized machinery would be an advantage to evolutionarily and geographically adjust to new circumstances. The skin color of people whose ancestors originated from lower latitude areas tends to be darker, whereas that of the people whose ancestors were from higher latitude regions tends to be lighter (Jablonski and Chaplin, 2000; Diamond, 2005). Therefore, we would propose that the production of ET-1, which is contributively engaged in developing and/or maintaining hyperpigmentation in a reciprocal manner with its receptor and with melanosomes would be intrinsically regulated to be further accelerated by the stimulation of melanosome incorporation so that an optimal protection against UV irradiation could be achieved. On the other hand, we have already discovered in a clinical study that the epidermal expression of SCF *in vivo* was significantly enhanced 3 days after irradiation with an early decrease whereas ET-1 was up-regulated 7 to 10 days after irradiation with a 2 minimal erythema dose (Hachiya et al., 2004). This delayed increase in ET-1 production compared to SCF expression might be explained by the aforementioned effect of melanosomes on keratinocytes.

In this study, we have verified that ET-1 signaling is consistently activated with both the significantly enhanced expression of ET-1 and ET-B in SLs and that treatment with ET-1 and/or an ET-B inhibitor substantially impacts the expression of melanogenic factors involved in the synthesis or the transport of melanin (melanosomes) in melanocytes. More importantly, ET-1 production in keratinocytes is accelerated according to the stimulation of melanosome incorporation, suggesting an elaborate role of increased ET-1 in producing and/or maintaining hyperpigmentation in harmony with an enhanced melanosome uptake into keratinocytes. These findings provide new insights for the fundamental understanding of regulatory mechanisms underlying the incidence and/or the maintenance of pigmented spots, and provide a basis to develop a more efficient strategy for their treatment. However, it has been previously reported that the reduced number of melanocytes in exposed skin exhibit an abnormal structure and function, resulting in the irregular distribution of melanin and in mottled skin with hypopigmented and hyperpigmented appearance according to the formation of SLs (Kushniruk, 1974). Since it is considered that some drastic alterations in melanocytes and probably in keratinocytes would occur in the course of SL development, further research is needed to fully understand the mechanisms underlying SL maintenance by focusing on the differences between SLs and UVB-induced pigmentation that gradually disappears within weeks (Videira et al., 2013).

## MATERIALS AND METHODS

### Materials

Human neonatal foreskins were provided by the National Disease Research Interchange (Philadelphia, PA, USA). Other chemicals were of reagent grade.

### Cell culture

Normal human epidermal keratinocytes (NHEKs) and normal human epidermal melanocytes (NHEMs) were isolated from human neonatal foreskins with modifications as described previously (Yoshida et al., 2007). NHEKs were preliminarily incubated either in EpiLife medium (Life Technologies, Carlsbad, CA, USA) or in supplemented-PCT epidermal keratinocyte medium (CnT-57; CELLnTEC Advanced Cell Systems Inc., Bern, Switzerland), both of which were supplemented as described previously (Murase et al., 2013). NHEMs were maintained in Medium 254 (Life Technologies) or in DermaLife^®^ M Melanocyte Culture Medium (Lifeline Cell Technology, Walkersville, MD, USA) as previously described (Murase et al., 2009). MNT-1 cells were preliminarily incubated in supplemented-RPMI-1640 medium as described previously (Murase et al., 2013). In co-culture experiments, NHEKs and NHEMs were seeded in 4-well culture slides pre-coated with Coating Matrix Kit (Life Technologies) at a density of 0.5×10^5^ cells/well and 0.1×10^5^ cells/well, respectively, in culture medium mixed with supplemented-PCT epidermal keratinocyte medium and supplemented-DermaLife^®^ M Melanocyte Culture Medium at a 3:1 ratio as described elsewhere (Murase et al., 2013).

### Human skin

Punch biopsy skin specimens, with or without SLs or uneven-toned areas, on the arms or shoulders of 41- to 60-year-old Caucasian, Asian or Hispanic females, were obtained at Stephens and Associates (Carrollton, TX, USA). Alternatively, skin biopsy samples from Caucasian subjects in their 30s and 40s with even- and/or uneven-toned skin were collected at RCTS, Inc. (Irvine, TX, USA) or at Colorado Dermatology Institute (Colorado Springs, CO, USA). The subjects were directed to avoid excess sun exposure at least a few weeks before their biopsies. The collections of skin tissues were approved by the Institutional Review Board of IntegReview Ltd. (Austin, TX, USA). This study was conducted according to the Declaration of Helsinki protocols and informed consent was obtained from each volunteer prior to the procedure.

### Melanosome incorporation into NHEKs

NHEKs were incubated with melanosomes isolated from MNT-1 cells as described previously (Murase et al., 2013; Ando et al., 2010).

### Quantitative Real-Time RT-PCR

Total RNA extracted from human epidermal sheet, whole skin, and cultured cells using RNeasy Micro Kit (Qiagen, Valencia, CA, USA) were used for the single-stranded cDNA synthesis by High-Capacity cDNA Reverse Transcription Kit (Life Technologies). Quantitative Real-Time PCR (qPCR) was performed with *Taq*Man Gene Expression Assay by using a StepOnePlus™ Real-Time PCR System (Life Technologies). The specific probes and primers which were used for each target gene were: *EDN1*, Hs00174961_m1; *EDNRB*, Hs00240747_m1; *KITLG*, Hs00241497_m1; *KIT*, Hs00174029_m1; *POMC*, Hs01596743_m1; *MC1R*, Hs00267167_s1; *TYR*, Hs00165976_m1; *WIPI1*, Hs00215872_m1 (Life Technologies). The expression of the genes of interest was normalized with that of *RPLP0* (ribosomal protein large P0, Hs99999902_m1) under relative standard curve method for each target. General conditions followed the MIQE guidelines (Bustin et al., 2009).

### Western blotting analysis

Samples were solubilized in 0.1 ml RIPA buffer (Thermo Fisher Scientific, Rockford, IL, USA) supplemented with a protease inhibitor cocktail (Roche, Rotkreuz, Switzerland) and homogenized using ultra-sonication. The resulting supernatants were collected as whole-cell lysates and their protein concentrations were determined using the BCA protein assay reagent (Pierce Biotechnology, Inc., Rockford, IL, USA). The whole-cell lysates were separated using 10 or 12% SDS-polyacrylamide gel electrophoresis and were then transferred to Millipore Immobilon^®^ FL PVDF Membranes (EMD Millipore, Billerica, MA, USA). Membranes were incubated with diluted primary antibodies specific for WIPI1 (Santa Cruz Biotechnology, Dallas, TX, USA; 1:500), Tyrosinase (1:20,000), Tyrp1 (Santa Cruz; 1:20,000), Rab27A (Santa Cruz; 1:500), Myosin Va (Santa Cruz; 1:500), PMEL17 (DAKO Inc., Carpinteria, CA, USA, clone HMB-45; 1:200 or 1:500), p62 (MBL International, Woburn, MA, USA; 1:2000) or LC3 (Cosmo Bio Co., Tokyo Japan; 1:2000 or MBL; 1:2000). The blots were subsequently washed and incubated with the appropriate diluted secondary antibodies [anti-mouse IgG peroxidase linked F(ab′)2 fragment, GE Healthcare UK Ltd., Buckinghamshire, UK; 1:10,000, anti-rabbit IgG peroxidase linked F(ab′)2 fragment, GE Healthcare; 1:10,000, or anti-goat IgG peroxidase linked F(ab′)2 fragment, Santa Cruz; 1:10,000]. Immunoreactive protein bands were visualized with ECL Prime western blotting detection reagents (GE Healthcare) and were quantified using an ODYSSEY Fc Imaging system (LICOR Inc., Lincoln, NE, USA). The amounts of protein loaded were normalized against β-actin using a monoclonal antibody specific for β-actin (Sigma-Aldrich Co., St Louis, MO, USA; 1:10,000) as an internal standard.

### Immunofluorescence microscopy analysis

Tissues were fixed upon slides with acetone, and then permeabilized with 0.1 µg/ml Triton X-100 in PBS. Tissues were incubated in 2.5% normal horse serum (Vector, Burlingame, CA, USA), followed by treatment with rabbit anti-ET-B antibody (Abcam Inc., Cambridge, MA, USA; 1:4000 dilution) and mouse anti-PMEL17 antibody (DAKO; 1:40). Tissues were incubated with Alexa Fluor^®^ 488 goat anti-rabbit IgG and Alexa Fluor^®^ 594 goat anti-mouse IgG (both from Life Technologies; 1:500), followed by nuclear staining with 4′6-diamidino-2-phenylindole (DAPI) (Life Technologies; 1:2000). Slides were mounted in Fluoromount-G^®^ (Southern Biotech, Birmingham, AL, USA). Co-cultured NHEKs and NHEMs in 4-well culture slides were fixed with 4% paraformaldehyde in PBS and were quenched by 50 mM NH_4_Cl in PBS. The cells were then permeabilized with 50 µg/ml digitonin in PBS and were then incubated in PBS containing 0.2% gelatin, followed by treatment with rabbit anti-p62 antibody (MBL; 1:500) and mouse anti-PMEL17 antibody (DAKO Inc.; 1:500). Cells were then incubated with Alexa Fluor^®^ 488 goat anti-rabbit IgG and Alexa Fluor^®^ 594 goat anti-mouse IgG (both from Life Technologies; 1:1000). Slides were mounted in ProLong^®^ Gold Antifade Reagent with DAPI (Life Technologies), and images were obtained with a Leica DM5500B digital microscope (Leica Microsystems, Bannockburn, IL, USA).

### Measurement of melanin contents in NHEMs

After solubilization in 200 µl Solvable™ (PerkinElmer, Waltham, MA, USA) of washed cells, melanin contents were measured using an absorbance meter (Microplate Reader Model 550; Bio-Rad Laboratories) at 405 nm.

### Fontana–Masson staining

The tissues were fixed with 10% buffered formalin, and were then embedded in paraffin. Melanin pigment was visualized using Fontana–Masson staining with an eosin counterstain as described previously (Hachiya et al., 2005). Images were obtained with a Leica DM5500B digital microscope (Leica Microsystems).

### Statistics

Significance of differences was calculated by Student's *t*-test, paired *t*-test, or ANOVA. A *P*-value <0.05 is considered statistically significant.

## References

[BIO011973C1] Abdel-Malek Z., Swope V. B., Suzuki I., Akcali C., Harriger M. D., Boyce S. T., Urabe K. and Hearing V. J. (1995). Mitogenic and melanogenic stimulation of normal human melanocytes by melanotropic peptides. *Proc. Natl. Acad. Sci. USA* 92, 1789-1793. 10.1073/pnas.92.5.1789 7878059 PMC42605

[BIO011973C2] Ahn G. Y., Butt K. I., Jindo T., Yaguchi H., Tsuboi R. and Ogawa H. (1998). The expression of endothelin-1 and its binding sites in mouse skin increased after ultraviolet B irradiation or local injection of tumor necrosis factor alpha. *J. Dermatol.* 25, 78-84. 10.1111/j.1346-8138.1998.tb02354.x 9563273

[BIO011973C3] Ando H., Niki Y., Yoshida M., Ito M., Akiyama K., Kim J.-H., Yoon T.-J., Lee J.-H., Matsui M. S. and Ichihashi M. (2010). Keratinocytes in culture accumulate phagocytosed melanosomes in the perinuclear area. *Pigment Cell Melanoma Res.* 23, 129-133. 10.1111/j.1755-148X.2009.00640.x 19761520

[BIO011973C4] Babiarz-Magee L., Chen N., Seiberg M. and Lin C. B. (2004). The expression and activation of protease-activated receptor-2 correlate with skin color. *Pigment Cell Res.* 17, 241-251. 10.1111/j.1600-0749.2004.00133.x 15140069

[BIO011973C5] Baumann L. (2007). Skin ageing and its treatment. *J. Pathol.* 211, 241-251. 10.1002/path.2098 17200942

[BIO011973C6] Boissy R. E., Huizing M. and Gahl W. A. (2006). Biogenesis of melanosomes. In *The Pigmentary System* (ed. Nordlund J. J., Boissy R. E., Hearing V. J., King R. A., Oetting W. S. and Ortonne J. P.), pp. 155-170. Oxford, UK: Blackwell Publishing.

[BIO011973C7] Bolognia J., Murray M. and Pawelek J. (1989). UVB-induced melanogenesis may be mediated through the MSH-receptor system. *J. Invest. Dermatol.* 92, 651-656. 10.1111/1523-1747.ep12696836 2497190

[BIO011973C8] Bustin S. A., Benes V., Garson J. A., Hellemans J., Huggett J., Kubista M., Mueller R., Nolan T., Pfaffl M. W., Shipley G. L. et al. (2009). The MIQE guidelines: minimum information for publication of quantitative real-time PCR experiments*.* *Clin. Chem.* 55, 611-622. 10.1373/clinchem.2008.112797 19246619

[BIO011973C9] Chakraborty A. K., Funasaka Y., Slominski A., Ermak G., Hwang J., Pawelek J. M. and Ichihashi M. (1996). Production and release of proopiomelanocortin (POMC) derived peptides by human melanocytes and keratinocytes in culture: regulation by ultraviolet B. *Biochim. Biophys. Acta* 1313, 130-138. 10.1016/0167-4889(96)00063-8 8781560

[BIO011973C10] Chung J. H., Hanft V. N. and Kang S. (2003). Aging and photoaging. *J. Am. Acad. Dermatol.* 49, 690-697. 10.1067/S0190-9622(03)02127-3 14512918

[BIO011973C11] Coelho S. G., Valencia J. C., Yin L., Smuda C., Mahns A., Kolbe L., Miller S. A., Beer J. Z., Zhang G., Tuma P. L. et al. (2015). UV exposure modulates hemidesmosome plasticity, contributing to long-term pigmentation in human skin. *J. Pathol.* 236, 17-29. 10.1002/path.4497 25488118 PMC4398603

[BIO011973C12] del Marmol V. and Beermann F. (1996). Tyrosinase and related proteins in mammalian pigmentation. *FEBS Lett.* 381, 165-168. 10.1016/0014-5793(96)00109-3 8601447

[BIO011973C13] Desai S. R. (2014). Hyperpigmentation therapy: a review. *J. Clin. Aesthet. Dermatol.* 7, 13-17. 25161755 PMC4142815

[BIO011973C14] Diamond J. (2005). Evolutionary biology: geography and skin colour. *Nature* 435, 283-284. 10.1038/435283a 15902239

[BIO011973C15] Fisher G. J., Kang S., Varani J., Bata-Csorgo Z., Wan Y., Datta S. and Voorhees J. J. (2002). Mechanisms of photoaging and chronological skin aging. *Arch. Dermatol.* 138, 1462-1470. 10.1001/archderm.138.11.1462 12437452

[BIO011973C16] Fukuda M. (2008). Regulation of secretory vesicle traffic by Rab small GTPases. *Cell Mol. Life Sci.* 65, 2801-2813. 10.1007/s00018-008-8351-4 18726178 PMC11131888

[BIO011973C17] Hachiya A., Kobayashi A., Ohuchi A., Takema Y. and Imokawa G. (2001). The paracrine role of stem cell factor/c-kit signaling in the activation of human melanocytes in ultraviolet-B-induced pigmentation. *J. Invest. Dermatol.* 116, 578-586. 10.1046/j.1523-1747.2001.01290.x 11286626

[BIO011973C18] Hachiya A., Kobayashi A., Yoshida Y., Kitahara T., Takema Y. and Imokawa G. (2004). Biphasic expression of two paracrine melanogenic cytokines, stem cell factor and endothelin-1, in ultraviolet B-induced human melanogenesis. *Am. J. Pathol.* 165, 2099-2109. 10.1016/S0002-9440(10)63260-9 15579452 PMC1618730

[BIO011973C19] Hachiya A., Sriwiriyanont P., Kaiho E., Kitahara T., Takema Y. and Tsuboi R. (2005). An in vivo mouse model of human skin substitute containing spontaneously sorted melanocytes demonstrates physiological changes after UVB irradiation. *J. Invest. Dermatol.* 125, 364-372. 16098048 10.1111/j.0022-202X.2005.23832.x

[BIO011973C20] Hachiya A., Sriwiriyanont P., Kobayashi T., Nagasawa A., Yoshida H., Ohuchi A., Kitahara T., Visscher M. O., Takema Y., Tsuboi R. et al. (2009). Stem cell factor-KIT signalling plays a pivotal role in regulating pigmentation in mammalian hair. *J. Pathol.* 218, 30-39. 10.1002/path.2503 19214986

[BIO011973C21] Halaban R., Langdon R., Birchall N., Cuono C., Baird A., Scott G., Moellmann G. and McGuire J. (1988). Basic fibroblast growth factor from human keratinocytes is a natural mitogen for melanocytes. *J. Cell Biol.* 107, 1611-1619. 10.1083/jcb.107.4.1611 2459134 PMC2115244

[BIO011973C22] Hattori H., Kawashima M., Ichikawa Y. and Imokawa G. (2004). The epidermal stem cell factor is over-expressed in lentigo senilis: implication for the mechanism of hyperpigmentation. *J. Invest. Dermatol.* 122, 1256-1265. 10.1111/j.0022-202X.2004.22503.x 15140230

[BIO011973C23] Ho H. and Ganesan A. K. (2011). The pleiotropic roles of autophagy regulators in melanogenesis. *Pigment Cell Melanoma Res.* 24, 595-604. 10.1111/j.1755-148X.2011.00889.x 21777401

[BIO011973C24] Ho H., Kapadia R., Al-Tahan S., Ahmad S. and Ganesan A. K. (2011). WIPI1 coordinates melanogenic gene transcription and melanosome formation via TORC1 inhibition. *J. Biol. Chem.* 286, 12509-12523. 10.1074/jbc.M110.200543 21317285 PMC3069453

[BIO011973C25] Hölzle E. (1992). Pigmented lesions as a sign of photodamage. *Br. J. Dermatol.* 127 Suppl. 41, 48-50. 10.1111/j.1365-2133.1992.tb16989.x 1390186

[BIO011973C26] Hyter S., Coleman D. J., Ganguli-Indra G., Merrill G. F., Ma S., Yanagisawa M. and Indra A. K. (2013). Endothelin-1 is a transcriptional target of p53 in epidermal keratinocytes and regulates ultraviolet-induced melanocyte homeostasis. *Pigment Cell Melanoma Res.* 26, 247-258. 10.1111/pcmr.12063 23279852 PMC3663331

[BIO011973C27] Imokawa G. and Mishima Y. (1982). Loss of melanogenic properties in tyrosinases induced by glucosylation inhibitors within malignant melanoma cells. *Cancer Res.* 42, 1994-2002. 6802485

[BIO011973C28] Imokawa G., Yada Y. and Miyagishi M. (1992). Endothelins secreted from human keratinocytes are intrinsic mitogens for human melanocytes. *J. Biol. Chem.* 267, 24675-24680. 1280264

[BIO011973C29] Imokawa G., Miyagishi M. and Yada Y. (1995). Endothelin-1 as a new melanogen: coordinated expression of its gene and the tyrosinase gene in UVB-exposed human epidermis. *J. Invest. Dermatol.* 105, 32-37. 10.1111/1523-1747.ep12312500 7615973

[BIO011973C30] Imokawa G., Yada Y. and Kimura M. (1996). Signaling mechanisms of endothelin-induced mitogenesis and melanogenesis in human melanocytes. *Biochem. J.* 314, 305-312. 10.1042/bj3140305 8660299 PMC1217041

[BIO011973C31] Imokawa G., Kobayashi T., Miyagishi M., Higashi K. and Yada Y. (1997). The role of endothelin-1 in epidermal hyperpigmentation and signaling mechanisms of mitogenesis and melanogenesis. *Pigment Cell Res.* 10, 218-228. 10.1111/j.1600-0749.1997.tb00488.x 9263329

[BIO011973C32] Imokawa G., Kobayashi T. and Miyagishi M. (2000). Intracellular signaling mechanisms leading to synergistic effects of endothelin-1 and stem cell factor on proliferation of cultured human melanocytes: cross-talk via trans-activation of the tyrosine kinase c-kit receptor. *J. Biol. Chem.* 275, 33321-33328. 10.1074/jbc.M004346200 10921922

[BIO011973C33] Iozumi K., Hoganson G. E., Pennella R., Everett M. A. and Fuller B. B. (1993). Role of tyrosinase as the determinant of pigmentation in cultured human melanocytes. *J. Invest. Dermatol.* 100, 806-811. 10.1111/1523-1747.ep12476630 8496620

[BIO011973C34] Jablonski N. G. and Chaplin G. (2000). The evolution of human skin coloration. *J. Hum. Evol.* 39, 57-106. 10.1006/jhev.2000.0403 10896812

[BIO011973C35] Jackson R. (2001). Elderly and sun-affected skin. Distinguishing between changes caused by aging and changes caused by habitual exposure to sun. *Can. Fam. Physician* 47, 1236-1243. 11421052 PMC2018527

[BIO011973C36] Kadono S., Manaka I., Kawashima M., Kobayashi T. and Imokawa G. (2001). The role of the epidermal endothelin cascade in the hyperpigmentation mechanism of lentigo senilis. *J. Invest. Dermatol.* 116, 571-577. 10.1046/j.1523-1747.2001.01296.x 11286625

[BIO011973C37] Kitamura R., Tsukamoto K., Harada K., Shimizu A., Shimada S., Kobayashi T. and Imokawa G. (2004). Mechanisms underlying the dysfunction of melanocytes in vitiligo epidermis: role of SCF/KIT protein interactions and the downstream effector, MITF-M. *J. Pathol.* 202, 463-475. 10.1002/path.1538 15095274

[BIO011973C38] Kushniruk W. (1974). Senescent skin. *Can. Fam. Physician* 20, 51-54. 20469067 PMC2274164

[BIO011973C39] Lerner A. B. and McGuire J. S. (1961). Effect of alpha- and beta-melanocyte stimulating hormones on the skin colour of man. *Nature* 189, 176-179. 10.1038/189176a0 13761067

[BIO011973C40] Levine B. and Klionsky D. J. (2004). Development by self-digestion: molecular mechanisms and biological functions of autophagy. *Dev. Cell* 6, 463-477. 10.1016/S1534-5807(04)00099-1 15068787

[BIO011973C41] Li L., Hu D.-N., Zhao H., McCormick S. A., Nordlund J. J. and Boissy R. E. (2006). Uveal melanocytes do not respond to or express receptors for alpha-melanocyte-stimulating hormone. *Invest. Ophthalmol. Vis. Sci.* 47, 4507-4512. 10.1167/iovs.06-0391 17003446

[BIO011973C42] Mackintosh J. A. (2001). The antimicrobial properties of melanocytes, melanosomes and melanin and the evolution of black skin. *J. Theor. Biol.* 211, 101-113. 10.1006/jtbi.2001.2331 11419954

[BIO011973C43] Manaka I., Kadono S., Kawashima M., Kobayashi T. and Imokawa G. (2001). The mechanism of hyperpigmentation in seborrhoeic keratosis involves the high expression of endothelin-converting enzyme-1alpha and TNF-alpha, which stimulate secretion of endothelin 1. *Br. J. Dermatol.* 145, 895-903. 10.1046/j.1365-2133.2001.04521.x 11899142

[BIO011973C44] Ménasché G., Ho C. H., Sanal O., Feldmann J., Tezcan I., Ersoy F., Houdusse A., Fischer A. and de Saint Basile G. (2003). Griscelli syndrome restricted to hypopigmentation results from a melanophilin defect (GS3) or a MYO5A F-exon deletion (GS1). *J. Clin. Invest.* 112, 450-456. 10.1172/JCI200318264 12897212 PMC166299

[BIO011973C45] Mishima Y. and Imokawa G. (1983). Selective aberration and pigment loss in melanosomes of malignant melanoma cells in vitro by glycosylation inhibitors: premelanosomes as glycoprotein. *J. Invest. Dermatol.* 81, 106-114. 10.1111/1523-1747.ep12542192 6409969

[BIO011973C46] Mizushima N. (2007). Autophagy: process and function. *Genes Dev.* 21, 2861-2873. 10.1101/gad.1599207 18006683

[BIO011973C47] Motokawa T., Kato T., Katagiri T., Matsunaga J., Takeuchi I., Tomita Y. and Suzuki I. (2005). Messenger RNA levels of melanogenesis-associated genes in lentigo senilis lesions. *J. Dermatol. Sci.* 37, 120-123. 10.1016/j.jdermsci.2004.10.009 15659332

[BIO011973C48] Murase D., Hachiya A., Amano Y., Ohuchi A., Kitahara T. and Takema Y. (2009). The essential role of p53 in hyperpigmentation of the skin via regulation of paracrine melanogenic cytokine receptor signaling. *J. Biol. Chem.* 284, 4343-4353. 10.1074/jbc.M805570200 19098008

[BIO011973C49] Murase D., Hachiya A., Takano K., Hicks R., Visscher M. O., Kitahara T., Hase T., Takema Y. and Yoshimori T. (2013). Autophagy has a significant role in determining skin color by regulating melanosome degradation in keratinocytes. *J. Invest. Dermatol.* 133, 2416-2424. 10.1038/jid.2013.165 23558403

[BIO011973C50] Okazaki K., Uzuka M., Morikawa F., Toda K. and Seiji M. (1976). Transfer mechanism of melanosomes in epidermal cell culture. *J. Invest. Dermatol.* 67, 541-547. 10.1111/1523-1747.ep12664554 787440

[BIO011973C51] Park H. Y., Russakovsky V., Ohno S. and Gilchrest B. A. (1993). The beta isoform of protein kinase C stimulates human melanogenesis by activating tyrosinase in pigment cells. *J. Biol. Chem.* 268, 11742-11749. 7685020

[BIO011973C52] Parra E. J. (2007). Human pigmentation variation: evolution, genetic basis, and implications for public health. *Am. J. Phys. Anthropol.* 134 Suppl. 45, 85-105. 10.1002/ajpa.20727 18046745

[BIO011973C53] Roméro-Graillet C., Aberdam E., Clément M., Ortonne J. P. and Ballotti R. (1997). Nitric oxide produced by ultraviolet-irradiated keratinocytes stimulates melanogenesis. *J. Clin. Invest.* 99, 635-642. 10.1172/JCI119206 9045865 PMC507845

[BIO011973C54] Rosdahl I. K. and Szabo G. (1978). Mitotic activity of epidermal melanocytes in UV-irradiated mouse skin. *J. Invest. Dermatol.* 70, 143-148. 10.1111/1523-1747.ep12258559 632619

[BIO011973C55] Schallreuter K. U., Kothari S., Chavan B. and Spencer J. D. (2008). Regulation of melanogenesis–controversies and new concepts. *Exp. Dermatol.* 17, 395-404. 10.1111/j.1600-0625.2007.00675.x 18177348

[BIO011973C56] Schauer E., Trautinger F., Köck A., Schwarz A., Bhardwaj R., Simon M., Ansel J. C., Schwarz T. and Luger T. A. (1994). Proopiomelanocortin-derived peptides are synthesized and released by human keratinocytes. *J. Clin. Invest.* 93, 2258-2262. 10.1172/JCI117224 8182158 PMC294380

[BIO011973C57] Scott G., Deng A., Rodriguez-Burford C., Seiberg M., Han R., Babiarz L., Grizzle W., Bell W. and Pentland A. (2001). Protease-activated receptor 2, a receptor involved in melanosome transfer, is upregulated in human skin by ultraviolet irradiation. *J. Invest. Dermatol.* 117, 1412-1420. 10.1046/j.0022-202x.2001.01575.x 11886502

[BIO011973C58] Searle A. G. (1990). Comparative genetics of albinism. *Ophthalmic Paediatr. Genet.* 11, 159-164. 10.3109/13816819009020974 2126367

[BIO011973C59] Seglen P. O. and Bohley P. (1992). Autophagy and other vacuolar protein degradation mechanisms. *Experientia* 48, 158-172. 10.1007/BF01923509 1740188

[BIO011973C60] Sharlow E. R., Paine C. S., Babiarz L., Eisinger M., Shapiro S. and Seiberg M. (2000). The protease-activated receptor-2 upregulates keratinocyte phagocytosis. *J. Cell Sci.* 113, 3093-3101. 10934047 10.1242/jcs.113.17.3093

[BIO011973C61] Sherratt M. J., Bayley C. P., Reilly S. M., Gibbs N. K., Griffiths C. E. and Watson R. E. (2010). Low-dose ultraviolet radiation selectively degrades chromophore-rich extracellular matrix components*.* *J. Pathol.* 222, 32-40. 10.1002/path.2730 20552716

[BIO011973C62] Sirsjö A., Karlsson M., Gidöf A., Rollman O. and Törmä H. (1996). Increased expression of inducible nitric oxide synthase in psoriatic skin and cytokine-stimulated cultured keratinocytes. *Br. J. Dermatol.* 134, 643-648. 10.1111/j.1365-2133.1996.tb06963.x 8733364

[BIO011973C63] Stenmark H. (2009). Rab GTPases as coordinators of vesicle traffic. *Nat. Rev. Mol. Cell Biol.* 10, 513-525. 10.1038/nrm2728 19603039

[BIO011973C64] Sturm R. A. (2009). Molecular genetics of human pigmentation diversity. *Hum. Mol. Genet.* 18, R9-R17. 10.1093/hmg/ddp003 19297406

[BIO011973C65] Takeda K., Takahashi N.-H. and Shibahara S. (2007). Neuroendocrine functions of melanocytes: beyond the skin-deep melanin maker. *Tohoku J. Exp. Med.* 211, 201-221. 10.1620/tjem.211.201 17347546

[BIO011973C66] Teraki E., Tajima S., Manaka I., Kawashima M., Miyagishi M. and Imokawa G. (1996). Role of endothelin-1 in hyperpigmentation in seborrhoeic keratosis. *Br. J. Dermatol.* 135, 918-923. 10.1046/j.1365-2133.1996.d01-1095.x 8977712

[BIO011973C67] Unver N., Freyschmidt-Paul P., Hörster S., Wenck H., Stäb F., Blatt T. and Elsässer H.-P. (2006). Alterations in the epidermal-dermal melanin axis and factor XIIIa melanophages in senile lentigo and ageing skin. *Br. J. Dermatol.* 155, 119-128. 10.1111/j.1365-2133.2006.07210.x 16792763

[BIO011973C68] Van Gele M., Dynoodt P. and Lambert J. (2009). Griscelli syndrome: a model system to study vesicular trafficking. *Pigment Cell Melanoma Res.* 22, 268-282. 10.1111/j.1755-148X.2009.00558.x 19243575

[BIO011973C69] Videira I. F., Moura D. F. L. and Magina S. (2013). Mechanisms regulating melanogenesis. *An. Bras. Dermatol.* 88, 76-83. 10.1590/S0365-05962013000100009 23539007 PMC3699939

[BIO011973C70] Wintzen M. and Gilchrest B. A. (1996). Proopiomelanocortin, its derived peptides, and the skin. *J. Invest. Dermatol.* 106, 673-678. 10.1111/1523-1747.ep12326950 8592078

[BIO011973C71] Yohn J. J., Morelli J. G., Walchack S. J., Rundell K. B., Norris D. A. and Zamora M. R. (1993). Cultured human keratinocytes synthesize and secrete endothelin-1. *J. Invest. Dermatol.* 100, 23-26. 10.1111/1523-1747.ep12349932 8423387

[BIO011973C72] Yoshida Y., Hachiya A., Sriwiriyanont P., Ohuchi A., Kitahara T., Takema Y., Visscher M. O. and Boissy R. E. (2007). Functional analysis of keratinocytes in skin color using a human skin substitute model composed of cells derived from different skin pigmentation types. *FASEB J.* 21, 2829-2839. 10.1096/fj.06-6845com 17475923

[BIO011973C73] Yoshida-Amano Y., Hachiya A., Ohuchi A., Kobinger G. P., Kitahara T., Takema Y. and Fukuda M. (2012). Essential role of RAB27A in determining constitutive human skin color. *PLoS ONE* 7, e41160 10.1371/journal.pone.0041160 22844437 PMC3402535

[BIO011973C74] Yoshimori T. (2004). Autophagy: a regulated bulk degradation process inside cells. *Biochem. Biophys. Res. Commun.* 313, 453-458. 10.1016/j.bbrc.2003.07.023 14684184

